# End-of-life Care in the Intesive Care Unit and Nursing Roles in Communicating with Families

**DOI:** 10.2478/jccm-2023-0013

**Published:** 2023-05-08

**Authors:** Anastasios Tzenalis, Helen Papaemmanuel, George Kipourgos, George Elesnitsalis

**Affiliations:** 1 University of Patras, Patra, Greece; 2 General Hospital of Thessaloniki Papageorgiou, Thessaloniki, Greece

**Keywords:** health professionals, family, death, communication, care, end of life care, intensive care unit, nurses

## Abstract

**Introduction:**

Professionals in Intensive Care Units face death, shifting their role from therapists to caregivers in end-of-life management. The nursing attitude and response to death has been shown to affect the quality of palliative care and end-of-life services that are interrelated services.

**Aim of the study:**

The aim of this research was to evaluate the professional attitude of nurses towards the care of the families of critically ill patients in the ICU, leading to the emergence of specific attitudes, relating them to their demographic and professional characteristics, with the aim of drawing conclusions for the improvement of quality in end-of-life care.

**Material and Methods:**

The sample of the study was 81 nurses from a large tertiary hospital. Participants completed the “Nurse Activities for Communicating with Families” (NACF) questionnaire. The questions are about ways in which nurses can help the patient’s family during the patient’s stay in the ICU.

**Results:**

The results revealed that the nurses took actions related to the information and psychological support of the patient’s family. On the contrary, they did not focus on the spiritual / religious needs of the patient and the needs of the family based on their cultural background.

**Conclusions:**

The professional treatment of staff is characterized by compassion and empathy, but it is necessary to train them on important issues related to diversity, including the religious, spiritual values and beliefs of patients and their relatives.

## INTRODUCTION

End of life care in Intensive Care Units (ICU) patients should focus on pain management, on patients’ dignity, and on the spiritual needs of the critically ill and their families. However, the abilities of ICU nurses to have an appropriate discussion with the patient’s relatives about the treatment or even the withdrawal of interventions, requires knowledge and communication skills, since palliative care includes issues of dealing with and treating physical symptoms, psychological and spiritual support, aiming at the quality death of the seriously ill patient [1]. The main concern must be the dignity of the patient based on proper communication, empathy, and avoiding conflicts with him and his family [2].The determinant for the quality of end-of-life care is to ensure dignity and respect for the patient, ensuring his personal wishes and the needs of both himself and his family environment [3].

The family is the one who is called upon to participate in this difficult situation, to accept the bad prognosis and the conditions for the limitation of life support and to make decisions for the care of their relative. Because of the seriousness of the condition in the ICU, patients usually have not the ability for their self-assessment of end-of-life care. [4]. Psychological support from the ICU team is important for the grief and bereavement of the family [5]. Family support is enhanced by proper communication since the patient’s family environment is forced to find themselves in a very stressful position in which they need to take crucial decisions on their relative’s end-of-life care [6].

The motivations for conducting the present study were, on the one hand, the fact that Nurses’ Activities for Communication with Families are not validated in the country and also that the ICU under study does not have IP support. But nevertheless, spiritual support of patients is provided by a priest, as well as specialized psychological support, when requested.

The aim of this research was to evaluate the professional attitude of nurses towards the care of the families of critically ill patients in the ICU, leading to the emergence of specific attitudes, relating them to their demographic and professional characteristics, with the aim of drawing conclusions for the improvement of quality in end-of-life care.

## MATERIALS AND METHODS

### Study design

Descriptive and correlational quantitative research was used for the purpose of this paper.

The research was based on the completion of anonymous questionnaires that focused on the evaluation of the nursing staff in their quality communication with the relatives of the critically ill patients in the ICU.

### Sample

The convenience sampling method was used to attract the sample in this research. The sample of the study was 81 nurses from a large tertiary hospital.

Inclusion criteria: Nurses and nurses’ assistants with more than one year of experience in an ICU.

Exclusion criteria: Nurses and nurses’ assistants with less than one year of experience in ICUs as well as other health professionals working in ICUs.

### Statistical analysis

The responses were categorized, and the resulting data were analyzed with the statistical program IBM SPSS Statistic 27.0.1. The level of statistical significance was set equal to α=0.05. The data analysis had a descriptive, correlational and qualitative character. The reliability of questions 1-18 was examined with the Cronbach’s index. It was found that there is a high degree of internal consistency (0.825).

### Research tool

Participants completed the “Nurse Activities for Communicating with Families” (NACF). The questions are about ways in which nurses can help the patient’s family during the patient’s stay in the ICU.

The specific questionnaire initially consists of questions that explore the demographic profile of the research participants (gender, age, marital status, educational level, years of service overall and in the ICU, and job position). It then includes 18 questions that explore the nurses’ activities in relation to the patient’s family, answered on a four-point Likert scale.

## RESULTS

Table 1 summarizes the demographic profile of the survey participants. As can be seen, the majority are women (69.1%), aged 40-49 (38.3%), with the same percentage of married and single (43.2%), graduates of higher education (61.7%), with 1-5 years of experience in the ICU (39.5%), graduate of higher technological education (TE) (79%).

**Table 1.** Demographic profile of respondents

	Frequency	%
**Gender**		
Male	25	30.9
Female	56	69.1
**Age**		
Up to 24 years old	7	8.6
25-29	14	17.3
30-34	17	21.0
35-39	7	8.6
40-49	31	38.3
50 and above	4	4.9
No Answer	1	1.2
**Marital status**		
Married	35	43.2
Single	35	43.2
Divorced	8	9.9
Widower	2	2.5
No Answer	1	1.2
**Educational level**		
Higher education	50	61.7
Private studies	7	8.6
Postgraduate studies	17	21.0
Ph.D	6	7.4
Higher education and private studies	1	1.2
**Previous experience in ICU**		
1-5	32	39.5
6-10	18	22.2
11-15	14	17.3
15 and above	17	21.0

### Descriptive statistics

In relation to respondents’ views on their attitudes and perceptions regarding end-of-life care and management of critically ill patients, it was shown that healthcare professionals were more likely to perform actions such as reassuring the family about that the patient’s comfort and relief has been ensured (M=1.37, TA=0.828), as well as informing them that they can talk to and touch their loved one (M=1.51, TA= 0.853). Also, high rates were recorded regarding adequate support for the family experiencing the end of their loved one life (M=1.85, TA=0.976), as well as regarding communication with the family about the patient’s illness and treatment (M=1.86, TA=1.034). Additionally, they were largely given information about logistics (M=1.95, TA=1.059), talked to the family about how they felt about the whole situation they were experiencing (M=1.95, TA=1.083), and the family was informed about what to expect from their communication with members of the nursing team (M=1.79, TA=0.918).

On the other hand, the interviewed nurses largely failed to develop a conversation with the family about their spiritual or religious needs (M=2.62, TA=0.799), as well as about what the patient considered important in life of (M=2.53, TA=0.963). Also, low rates were recorded regarding the investigation of any disagreements between the family regarding the care plan (M=2.61, TA=0.771), as well as regarding whether there were specific needs of the family due to particular cultural factors (M =2.49, TA=0.937). In table 2, the descriptive measures (means and standard deviations) of respondents’ views on each question are shown.

**Table 2.** Descriptive measures of respondents’ attitudes and perceptions regarding end-of-life care and management of critically ill patients

Questions		N	Median	Standard deviation
**Q1**	Did you explain the patient’s medical equipment and treatments to the family?	81	1.95	1.059
**Q2**	Have you told the family what to expect from their communication with members of the nursing team?	81	1.79	0.918
**Q3**	Did you discuss the spiritual or religious needs of the patient’s family?	81	2.62	0.799
**Q4**	Did you take steps to meet the family’s spiritual or religious needs?	81	2.38	1.019
**Q5**	Did you talk to the family about their specific needs based on their culture?	81	2.49	0.937
**Q6**	Did you take action to meet family needs identified in their own culture?	81	2.37	1.006
**Q7**	Did you talk to the family about what the patient considered important in his life?	81	2.53	0.963
**Q8**	Did you talk to the family about the patient’s illness and treatment?	81	1.86	1.034
**Q9**	Have you talked to the family about how they feel about this whole situation they are going through?	81	1.95	1.083
**Q10**	Did you discuss with the family reminiscing moments of the patient’s life?	81	2.47	0.896
**Q11**	Have you talked to the family about being able to talk and touch their loved one?	81	1.51	0.853
**Q12**	Have you discussed with the family what the patient would like if they could participate in the decision about their treatment choice?	81	2.43	0.921
**Q13**	Have you secured a private place or room for the family so they can talk to each other?	80	2.26	0.882
**Q14**	Have you talked with the family about any disagreements between the family about the care plan?	80	2.61	0.771
**Q15**	Have you talked to the family about the changes in the patient’s care plan?	80	2.33	0.978
**Q16**	Did you support the decision(s) the family made regarding the patient’s care?	80	2.15	1.032
**Q17**	Have you reassured the family that the patient’s comfort and relief has been ensured?	81	1.37	0.828
**Q18**	Did you offer adequate support to the family experiencing the end of their patient’s life?	81	1.85	0.976

### Inductive statistics

The existence of a difference in the mean attitudes and perceptions of health professionals based on their demographics was investigated, and to determine the type of statistical tests used, a test of normality of the data was performed with the Kolmogorov-Smirnov test. As the data do not follow a normal distribution (p<0.05), non-parametric tests were therefore used.

First, it was examined whether there was a difference in the means of participants’ attitudes and perceptions based on gender. The Mann-Whitney test was used for the test, and the results showed that there is no statistically significant difference.

It is then examined whether there is a difference in the averages of participants’ attitudes and perceptions based on age. The Kruskal-Wallis test was used for testing. The results are listed in Table 3. A statistically significant difference is observed in terms of whether they talked to the family about the patient’s illness and treatment (p<0.05), with those aged 40-49 having a higher mean rank and those aged 30 -34 to have lower.

**Table 3.** Mean Difference of Participants’ Attitudes and Perceptions by Age

Questions	Age	N	Mean Rank	p-value
Q 8 Did you talk to the family about the patient’s illness and treatment?	Up to 24 years old	7	47.93	**0.042**
	25-29	14	36.96	
	30-34	17	31.44	
	35-39	7	51.36	
	40-49	31	43.97	
	50 and above	4	32.50	
	Total	80		

Then, through the Kruskal-Wallis test, it was studied whether there is a difference in the averages of the attitudes and perceptions of the participants based on the factor of marital status. The results are listed in Table 4, from where it is observed that there is a statistically significant difference as to whether the nursing staff developed a discussion with the family regarding moments of recollection of the patient’s life. Divorced/widowed nurses were found to have the lowest mean.

**Table 4.** Mean difference of participants’ attitudes and perceptions by marital status

Questions	Marital Status	N	Mean Rank	p-value
Q 10 Did you discuss with the family reminiscing moments of the patient’s life?	Married	35	40.26	**0,025**
	Single	35	45.11	
	Divorced	8	29.00	
	Widower	2	10.00	
	Total	80		

Then, a Kruskal-Wallis test was performed, where the research hypothesis that the educational level affects the averages of the attitudes and perceptions of the participants was studied. The only statistically significant difference was recorded in the questions about whether they talked to the family about specific needs based on their culture and whether they talked to the family about the patient’s illness and treatment, where having a doctorate degree was shown to be a negative factor, causing lower average rank.

The last demographic factor that was studied for whether or not it influenced the averages of participants’ attitudes and perceptions was years of professional experience in the ICU. The Kruskal-Wallis test was used for the test and the results showed that there is a statistically significant difference as to whether they informed the family about their ability to talk and touch their loved one. Nurses with 11-15 years of professional experience appeared to have the lowest average ranking.

## DISCUSSION

Most of the nurses in the present research carried out actions related to the information and psychological support of the patient’s family. This finding is in line with what is reported in the international literature regarding the care of patients in ICUs and the communication of nurses with their families [1, 6–11].

A second important aspect of the research is that health professionals did not focus on the spiritual/religious needs of the patient on the one hand and on the needs of the family on the other hand based on their cultural background. Indeed, although many ICU palliative care and end-of-life care systems consider the spiritual and religious needs of the family to be an important concern, it has been revealed in studies of 68 US ICUs [12], that the quality of end-of-life has a deficient provision of spiritual support. Also, in a study done in a Dutch ICU in 2017 and in which almost all ICUs have a spiritual caregiver, nurses understand the importance of spiritual care, but they themselves do not consider themselves capable or competent in that they think they can spiritual caregivers do it best [13].

Greece is becoming a country with Orthodox Christianity as its main religion, where spiritual needs are often identified with religious needs and the ritual can be limited to Eucharist, holy blessing oil and holy water. These needs are requested by the family, as usually the ICU patient cannot communicate with the nurses [14].

On the contrary, in other countries such as the USA, which is a multi-ethnic population, contact with other races and religions is common. American values emphasize autonomy and individual rights but also the possibility of choices due to different nationalities and culturally different groups [15]. Respondents in our survey did not mention the spiritual-religious values and needs of patients and families, although it is an important parameter for end-of-life care.

Additionally, the demographic characteristics of respondents greatly influence the opinions they expressed. More specifically, it was found that gender is not a factor that leads to statistically significant differences between the attitudes and behaviors of health professionals, a finding that is supported by previous studies [16, 17]. On the other hand, there are studies that have shown that demographic factors such as age [16], marital status [18], educational level [19, 20] years of professional experience [17, 21], influence individual attitudes and behaviors of health professionals.

It appears that the nurses in our research were largely confident in ensuring the patient’s comfort and symptom relief and were honestly able to assure the family of this, undertaking a very important aspect of end-of-life care, as in many surveys such as in Spain, it was pointed out how important it is for the family to ensure the comfort of their patient, and the nurses reached the rate of 87.9% to always or almost always succeed [22].

Regardless of the years of service, the respondents vary in almost the same percentages of those who support the family’s decisions as those who do not, a finding that has already been reported in another study [16]. Possibly, because they do not know how important it is for the family to have the support of their decisions by health professionals. In addition, it also depends on the years of experience of the nurses that determine the attitude and management towards death itself and religious beliefs.

Despite the fact that a large percentage of the sample discussed with the family how they feel about this whole situation they are experiencing, at the same time a percentage of respondents who did not provide information about any disagreements between the family or what the patient considered important, something that is mentioned in a 2016 study [23]. Possibly due to the inability of nurses to recognize the family’s points of disagreement, such as delaying decisions, questioning and wavering of their decisions, but also due to the lack of ability to resolve any conflicts.

An important aspect is to avoid discussing with the family the wishes of the patient to participate in both the decision and the choice of his treatment. This fact is also highlighted in other research as talking with the patient about death is a taboo for many cultures and religions [24], it is painful and requires a lot of specialized knowledge.

Finally, it is considered certain that the possible future development of ICU palliative care policies in the country will lead to important results, as according to the conclusions of a systematic review of 37 studies [25], it was shown that palliative care interventions carried out in the ICU reduced hospital and ICU length of stay, while they did not change either in-hospital mortality or family satisfaction. The interventions also improved the quality, quantity, and content of communication and reduced symptoms of distress and anxiety in family members.

## CONCLUSIONS

Although the nursing training in end-of-life care is incomplete, there is empathy and understanding of the emotions of the family environment by health professionals so that they know how important it is to inform and provide psychological support to the patients’ family. Despite the absence of protocols in their intensive care units, health professionals understand the anxiety of relatives about whether the comfort and relief of the patient is covered, since this is their main concern. Based on the findings of this research, the need for further training of the staff in issues related to cultural diversity, including the religious and spiritual values and beliefs of patients and their relatives in the end-of-life care, is demonstrated.
